# Preoperative quadriceps malalignment is associated with poor outcomes after knee replacement which are avoided by external rotation of the femoral component

**DOI:** 10.1002/ksa.12544

**Published:** 2024-12-12

**Authors:** Simon Talbot, Rachel Zordan, Francesca Sasanelli, Matthew Sun

**Affiliations:** ^1^ Department of Orthopaedic Surgery Western Health Melbourne Australia; ^2^ Faculty of Medicine, Dentistry and Health Sciences University of Melbourne Parkville Victoria Australia; ^3^ Education and Learning St. Vincent's Hospital Melbourne Victoria Australia

**Keywords:** arthroplasty, knee, knee replacement, patella, quadriceps

## Abstract

**Purpose:**

Lateralisation of the proximal apex of the quadriceps tendon relative to the mechanical axis or external rotation relative to the femoral shaft can be accurately measured and is strongly associated with patella maltracking. The aim of this study was to first assess the association between preoperative quadriceps tendon alignment (QTA) and the patient‐reported outcomes (PROMs) of total knee replacement, and second, determine the influence of component position on outcomes in patients with preoperative quadriceps tendon malalignment (QTM).

**Methods:**

A retrospective analysis of prospectively collected data was performed. All patients had preoperative and postoperative CT scans performed. PROMs were collected preoperatively and at 1 year postoperatively. QTA was measured by the quadriceps tendon axial angle (QTAx). The preoperative and postoperative coronal and axial alignment were measured. Femoral component rotation was measured relative to the preoperative posterior condyles.

**Results:**

Analysis was conducted on 388 cases and the mean preoperative QTAx was 6.2° externally rotated (standard deviation 12.0°). QTM (QTAx > 14°) was identified in 76 (19.8%) patients. The diagnosis of QTM was associated with reduced patient outcomes including Forgotten Joint Score (60.2 vs. 51.2, *p* = 0.008), EuroQol Visual Analogue Scale (81.3 vs. 75.7, *p* = 0.009), KOOS‐12 (80.3 vs. 73.3, *p* = 0.001) and reduced PASS percentages for all KOOS subscales. In patients with preoperative QTM, femoral component external rotation >2° was associated with improved PROMs when compared to patients with <2° of femoral rotation. This included a clinically significant difference in the improvement of KOOS‐12 (11.7 points, *p* = 0.013) and improved PASS percentages in all KOOS subscales. There was no association between coronal alignment or tibial axial alignment and outcomes.

**Conclusions:**

Quadriceps malalignment is a common cause for poorer patient outcomes following total knee replacement. This can be avoided by externally rotating the femoral component to accommodate the deformity in the extensor mechanism.

**Level of Evidence:**

Level III, case‐control study.

**Abbreviations**
AEA: anatomical epicondylar axis
FJS: Forgotten Joint Score
KOOS: Knee Injury and Osteoarthritis Outcome Score
LDFA: lateral distal femoral angle
LFPFJOA: lateral facet patellofemoral osteoarthritis
MCID: minimal clinical important difference
MPTA: medial proximal tibial angle
PASS: patient acceptable symptom state
PROMs: patient‐reported outcomes measures
Q‐angle: quadriceps angle
QTA: quadriceps tendon alignment
QTAx: quadriceps tendon axial angle
QTCA: quadriceps tendon coronal angle
QTM: quadriceps tendon malalignment

## INTRODUCTION

Patella maltracking is associated with poor outcomes following total knee arthroplasty (TKA) [1, 2, 9, 22]. It is caused by extensor mechanism malalignment relative to the flexion–extension axis of the knee [21, 30]. Patellofemoral alignment is highly variable in the normal population [13]. Identifying patients who have pathological extensor mechanism malalignment before TKA is important in determining patients at risk of poor outcomes and in developing individualised alignment techniques for these patients.

The association between quadriceps alignment and patella maltracking has been examined [19]. Traditionally, the alignment of the quadriceps has been assumed to correlate with the quadriceps angle (Q‐angle), measured from the patella to the anterior inferior iliac crest [18]. However, the Q‐angle has a poor correlation with patellofemoral kinematics [4, 10]. The Q‐angle only measures the bony attachments of the rectus femoral portion of the quadriceps muscle. It is likely that the bony attachments of the other three muscle bellies of the quadriceps, which attach to the femur and not the pelvis, are not aligned parallel to the rectus femoris. Therefore, the true force vector of the quadriceps complex may be significantly different from the Q‐angle. In support of this concept, the rotational alignment of the quadriceps muscle has been shown to rotate around the shaft of the femur in patients with patella instability [20].

A recent publication by Talbot et al. measured the alignment of the quadriceps tendon alignment (QTA) relative to the centre of the knee joint in a normal population and describes a wide range of variability in the anatomy [27]. The Quadriceps Tendon Coronal Angle (QTCA), the angle of the quadriceps tendon relative to the mechanical axis, varies from 14° varus to 7° valgus. The quadriceps tendon axial angle (QTAx), the angle between the apex of the quadriceps tendon and the centre of the shaft of the femur, varies from 44° externally rotated to 42° internally rotated [27]. Additionally, the alignment of the quadriceps tendon is not associated with any other measure of bony anatomy including femoral torsion, trochlear groove or posterior condyle alignment. This indicates that malalignment of the quadriceps tendon is indicative of an isolated quadriceps muscle deformity [27]. The QTAx in a group with lateral facet patellofemoral joint osteoarthritis (LFPFJOA) (*n* = 25) was 17.3° compared to 3.3° in a nonarthritic group (*n* = 25). The strength of the association indicates that Quadriceps Tendon Malalignment (QTM) is the predominant anatomical deformity associated with the development of patella maltracking causing severe LFPFJOA [27]. In confirming the relationship between lateralisation of the quadriceps tendon and development of LFPFJOA, Talbot et al. demonstrated the clinical relevance of QTM. Previous work by Mizuno et al. showed in a cadaveric study that altering the alignment of the quadriceps mechanism creates changes in the orientation of the patella and patellofemoral contact pressures during flexion [21], supporting the findings of Talbot et al.

The objective of this study is to explore the relationship between QTA, femoral component position and patient‐reported outcome measures (PROMs) in patients undergoing TKA. It aims to (i) identify an association between QTM and patient outcomes following TKA and (ii) using a subgroup of patients with QTM, determine the relationship between femoral component position and PROMs. The hypotheses are (i) preoperative QTA influences TKA outcomes and (ii) in patients with preoperative QTM, outcomes of TKA are influenced by femoral component axial rotation.

## MATERIALS AND METHODS

Prospectively collected preoperative and postoperative PROMs data were linked to retrospectively collected preoperative measurements of patient anatomy and postoperative measurements of component position. The analysis was performed on the data of patients undergoing TKA by a single surgeon over a 4‐year period (November 2018–November 2022). All patients undergoing TKA with a single prosthesis (Saiph; Matortho) were included. Exclusion criteria included absent or inadequate preoperative CT scans, previous fractures with malunion, previous tibial or femoral osteotomy, missing preoperative or postoperative PROMs. Of 574 cases, 144 were excluded due to missing preoperative or postoperative PROMs data and a further 42 were excluded due to absent or inadequate CT scans, resulting in 388 cases for analysis.

CT images consist of 1.25 mm slices through the hip, knee and ankle (GE Optima 660 Brightspeed, 128‐slice scanner). The scans were segmented and analysed using mediCAD® 2.1 3D Knee software (mediCAD Hectec GmbH, Altdorf/Landshut). Scans were first oriented to a consistent reference frame. The axial plane was oriented to a tangential line across the posterior condyles of the femur. The coronal plane was oriented to the plane of the mechanical axis of the femur from the hip centre to the centre of the distal point of the trochlear groove. The sagittal plane was oriented to the mechanical axis of the femur from the hip centre to the knee centre identified as the distal point of the trochlear groove at the apex of the intercondylar notch.

**Figure 1:**
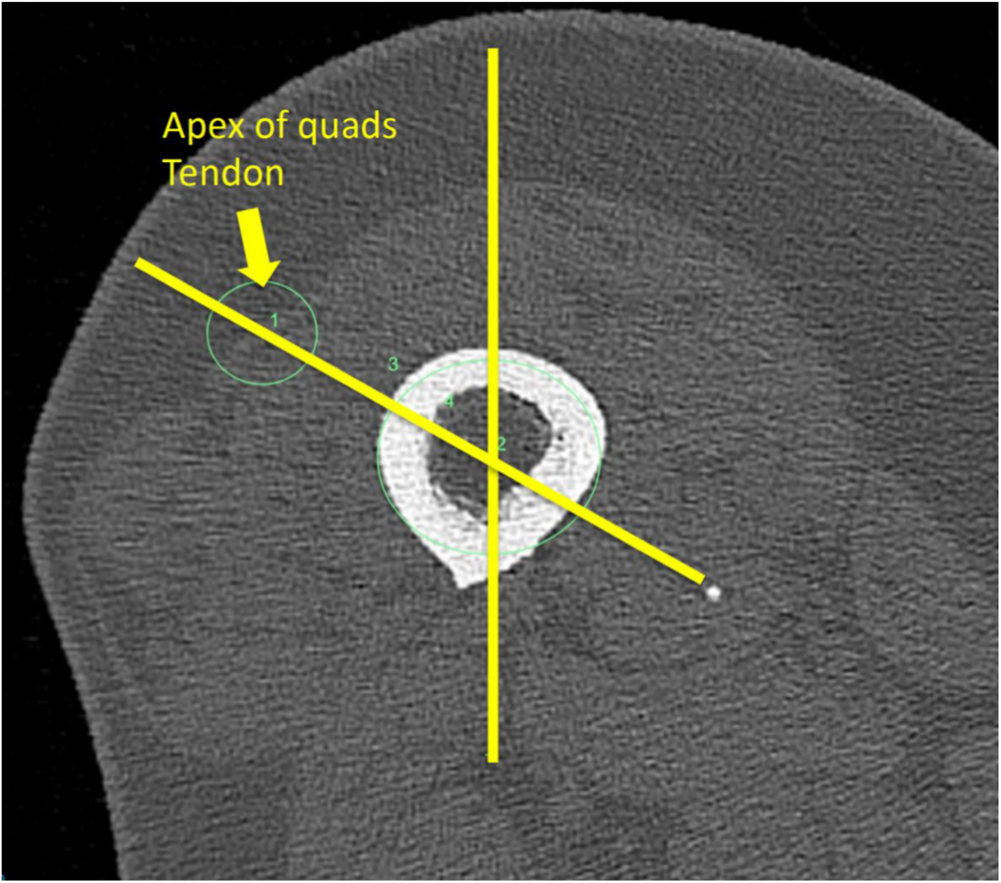
Quadriceps tendon axial angle. Posterior condyles are horizontal. Quadriceps tendon is lateralised relative to the femoral shaft.

The published QTAx technique was used to measure preoperative QTA [27]. QTAx was measured by identifying the centre of the apex of the QT using the centre point of a circle around the tendon. The centre of the axial cross‐section of the femur on the same axial slice was identified using the centre of the circle. The angle was measured from a vertical line perpendicular to the posterior condyle to a line between the centres of the two measured circles. A positive value was recorded when the apex of the quadriceps tendon was lateral to the centre of the femur (see Figure 1).

### Surgical technique

The surgical technique was standardised in all cases. Preoperative planning was based on CT scan results and the alignment technique personalised for each patient. The alignment technique was as follows: the starting point was neutral coronal alignment which was adjusted toward constitutional alignment based on ligament balance and prearthritic anatomy. Femoral rotation was adjusted between 0° and 5° of external rotation relative to the posterior condyles based on the position of the surgical epicondylar axis, the trochlear groove, the presence of preoperative patella maltracking and the intraoperative ligament balance. QTA was not measured before surgery. Adjustments to bony alignment, most commonly the tibial cut, were performed when necessary to minimise the requirement for ligament releases. No tourniquet was used. Medial parapatellar approach was performed. In all cases, the patella was resurfaced with a dome button. A lateral facetectomy was performed to remove any uncovered lateral patella bone. Femoral sizing was posterior referenced, and the component was positioned with a flush anterior osteotomy to avoid anterior overstuffing and to allow downsizing and lateralisation of the femoral component. Local anaesthetic infiltration, tranexamic acid and thromboprophylaxis were used. Postoperatively, mobilisation of the patient commenced on Day 1 (morning), with full weight bearing and full range of motion. A CT scan was performed between Days 2 and 4.

### Measurement technique

Component position was determined from postoperative CT scans. The scans were segmented and analysed using the mediCAD® 2.1 3D Knee software (mediCAD Hectec GmbH, Altdorf/Landshut). Coronal alignment of the femoral and tibial components were measured relative to mechanical axis and changes in lateral distal femur angle (LDFA) and medial proximal tibial angle (MPTA) were calculated by comparing the preoperative anatomy. Tibial component rotation was measured relative to Akagi's Line [1]. The change in posterior condyle angle was measured by comparing the preoperative posterior condylar angle relative to the Anatomical Epicondylar Axis (AEA) to the postoperative angle between the posterior condyles of the component and the AEA.

Twenty cases were randomly selected and measured by the senior author and an orthopaedic resident to determine inter‐ and intraobserver reliability. Both assessors have extensive experience in performing these measurements. Upon confirmation of inter‐ and intraobserver reliability, the senior author measured all cases.

### Patient‐reported outcome measures

PROMs data were collected using an online questionnaire sent via email and completed by patients pre‐ and postoperatively (12 months). The questionnaire included the Knee Injury and Osteoarthritis Outcome Score (KOOS) [24], Forgotten Joint Score (FJS) [3] and EuroQol Visual Analogue Scale (EQ‐VAS) [12].

The KOOS [24] is a knee‐specific instrument used to measure functional recovery and quality of life (QoL) in patients following TKA [29]. The KOOS includes five scales: pain, symptoms, activities of daily living, sports and recreation, and knee‐related quality of life [24]. All subscales are scored separately from 0 to 100 points, with 0 indicating extreme knee problems and 100 indicating no knee problems. Patient acceptable symptom state (PASS) cut‐points for KOOS subscales were greater than 84.5 points for pain, 80.5 points for symptoms, 83.0 points for activities of daily living and 66.0 points for quality of life [5]. KOOS sport PASS data cut‐point is not available for patients undergoing TKA. A KOOS‐12 score was calculated from the relevant items [11], with the Minimal Clinical Important Difference (MCID) for the KOOS‐12 at 11.1 points [7].

The FJS is a questionnaire based on the assumption that the ability to forget an artificial joint is the goal following joint arthroplasty. The FJS uses a 5‐point Likert response format, consisting of 12 questions, each measuring the awareness of the artificial joint in daily activities. FJS score ranges 0–100 points with a higher score corresponding to a better outcome.

The EQ‐VAS is a stand‐alone component of the EQ‐5D‐5L index [12], in which a patient self‐reports the impression of their general health and functionality. Patients rate their general health from 0 to 100, with higher scores indicating better function. EQ‐VAS has been evaluated and demonstrated validity in an Australian population undergoing TKA [17].

### Data analysis

Sample size calculation was performed to adequately power comparative analysis between patients with QTM based on the level of femoral component rotation. A minimum sample size of 70 patients with QTM had a 95% chance of detecting such a difference, with an alpha level of 0.05. Based on the assumption that 20% of patients would be assessed as having QTM, a total sample size of 350 cases was required. Interobserver and intraobserver reliability was calculated using the intraclass correlation coefficient. The strength of the relationship was assessed as low/weak (*r* < 0.25), fair (*r* = 0.25 to < 0.50), good (*r* = 0.50–0.75) or excellent (*r* > 0.75). A KOOS‐12 change score was calculated by subtracting the KOOS‐12 preoperative score from the postoperative KOOS‐12 score. Independent sample *t* tests were conducted comparing patient groups (QTM to non‐QTM) and a subgroup of patients with QTM. The subgroup with QTM was divided into two groups using the femoral component rotation median distribution score. Pearson correlation coefficients were calculated for femoral rotation and PROMs. The results are reported as means, ± standard deviation (SD), range and 95% confidence intervals (95%CI). Data were analysed using IBM SPSS Statistics software (version 29.2.0).

## RESULTS

Excellent intra‐ and interobserver reliabilities were demonstrated between CT measurements with *r* > 0.8.

### Aim 1

The QTAx preoperative measurement mean was 6.2° (SD 12.0°, range −33° to 54°). Based on previous research [27], an angle of >14° is a clinically relevant level of QTM. Seventy‐six patients (19.6%) fulfilled the criteria for QTM. There were no significant differences in age or sex between the two groups. Demographic data are shown in Table 1.

**Table 1:** Demographic data of patients with non‐QTM and QTM.

	Non‐QTM (QTAx ≤ 14°) (*n* = 312)	QTM (QTAx > 14°) (*n* = 76)	*p* value
Sex (female %)	52.8	43.2	>0.05
Age (years)	69.9	67.4	>0.05

Abbreviation: QTM, quadriceps tendon malalignment.

Compared to the non‐QTM group, the QTM group reported lower scores across all PROMs indicating poorer postoperative outcomes. This difference was statistically significant in all KOOS scales (pain, symptoms, activities of daily living, sport, QoL), the FJS, and the EQ‐VAS. Comparative analysis of patients with or without QTM is shown in Table 2. The comparison of KOOS scale PASS percentages is shown in Table 3. There was no association between internally rotated QTAx (medialised quadriceps tendon) and patient outcomes.

**Table 2:** Comparison of PROMs in patients with non‐QTM and QTM (QTAx > 14°).

	Non‐QTM (*n* = 312)		QTM (*n* = 76)		Mean difference	95% CI	*p* value
	Mean	SD	Mean	SD			
**KOOS**							
Pain	87.0	14.2	80.4	17.0	6.6	2.7–10.5	0.001
Symptoms	80.6	15.0	74.4	17.6	6.2	2.1–10.3	0.001
ADL	88.5	13.2	82.1	16.8	6.5	2.8–10.1	0.001
Sport	59.2	26.0	51.2	20.5	8.0	1.2–14.8	0.011
QoL	72.0	20.7	65.2	20.9	6.9	1.5–12.3	0.006
KOOS‐12	80.3	16.1	73.3	17.9	7.0	2.7–11.3	0.001
**FJS**	60.2	28.3	51.2	29.9	9.0	1.7–16.1	0.008
**EQ‐VAS**	81.3	17.7	75.7	21.4	5.6	0.9–10.2	0.009

Abbreviations: ADL, activities of daily living; CI, confidence interval; EQ‐VAS, EuroQol Visual Analogue Scale; FJS, Forgotten Joint Scale; KOOS, Knee Injury and Osteoarthritis Outcome Score; PROM, patient‐reported outcome; QoL, quality of life; QTAx, quadriceps tendon axial angle; QTM, quadriceps tendon malalignment; SD, standard deviation.

**Table 3:** KOOS PASS percentage in patients with QTM (QTAx > 14°) compared to patients with non‐QTM.

	Non‐QTM (*n* = 312)	QTM (*n* = 76)	*p* value
**KOOS PASS**	%	%	
Pain (>84.5)	65.5	50.6	0.016
Symptoms (>80.5)	56.1	39.5	0.009
ADL (>83.0)	75.2	57.1	0.002
QoL (>66.0)	64.3	50.6	0.027

Abbreviations: ADL, activities of daily living; KOOS, Knee Injury and Osteoarthritis Outcome Score; PASS, patient acceptable symptom state; QoL, quality of life; QTAx, quadriceps tendon axial angle; QTM, quadriceps tendon malalignment.

### Aim 2

**Figure 2:**
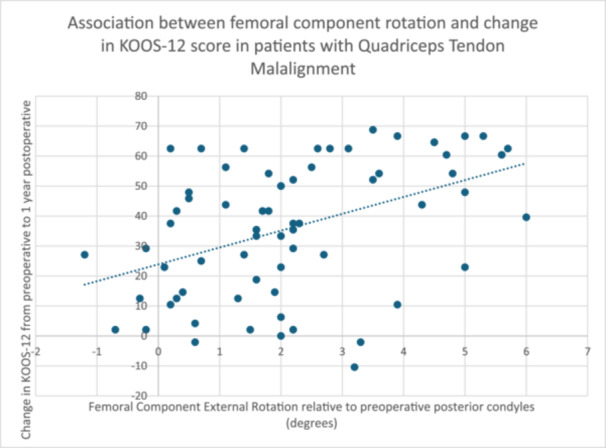
Association between femoral component rotation and change in Knee Injury and Osteoarthritis Outcome Score‐12 score in patients with quadriceps tendon malalignment.

Using data from patients identified with QTM (*n *= 76), analysis was undertaken to assess the effect, if any, of femoral component position on PROMs. The association between femoral component rotation and change in KOOS‐12 score in patients with QTM is shown in Figure 2. There was no significant association between postoperative LDFA, MPTA, tibial component rotation, change in LDFA or change in MPTA in patients with QTM.

The correlations between outcomes and femoral component rotation are reported in Table 4.

**Table 4:** Pearson's correlations between femoral component rotation and outcomes in patients with QTM.

	Correlations with femoral component rotation in cases with QTM	*p* value
FJS	0.379	<0.001
KOOS‐12	0.397	<0.001
EQ‐5D	0.046	NS
Change in KOOS‐12	0.345	<0.001

Abbreviations: FJS, Forgotten Joint Scale; KOOS, Knee Injury and Osteoarthritis Outcome Score; QTM, quadriceps tendon malalignment.

Patients with QTM who reported a KOOS‐12 > 75 (*n* = 40) had a mean femoral component external rotation of 2.9° (SD 1.4°) compared to a mean of 1.4° (SD 1.8°) in patients with a KOOS‐12 < 75 (*p* < 0.001, *n* = 36).

**Figure 3:**
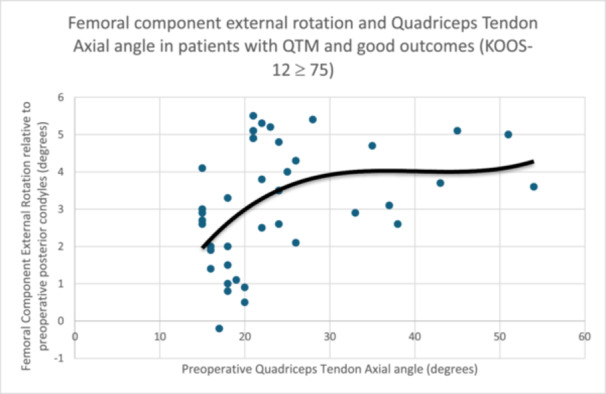
Femoral component external rotation and quadriceps tendon axial angle in patients with quadriceps tendon malalignment and Knee Injury and Osteoarthritis Outcome Score‐12 score ≥75.

There was a positive correlation between femoral component rotation and QTAx angle in patients who achieved a good outcome (KOOS‐12 > 75) after TKA (*r *= 0.536, *p *< 0.001). This indicates that patients with more severe QTM require greater femoral component external rotation to optimise outcomes. This is shown in Figure 3.

Patients with QTM were divided into two groups based on the median femoral component rotation. Due to postoperative metal artefact obscuring landmarks, the femoral component rotation was measured in 70 of 76 patients. Patients who had ≤2° of femoral component external rotation had a mean KOOS‐12 of 70.0 (SD 16.8) compared to patients with >2° of femoral component external rotation who scored a mean KOOS‐12 of 79.3 (SD 18.1; *p* = 015). Patients who had ≤2° of femoral component external rotation had a mean change in KOOS‐12 of 31.3 (SD 21.3) compared to patients with >2° of femoral component external rotation who had mean change in KOOS‐12 of 43.0 (SD 21.9). Therefore, the difference in the change in KOOS‐12 between the two groups was 11.7 points (CI = 1.4–22.0, *p* = 0.013).

Remaining PROMs and KOOS subscores are summarised in Table 5. There was no difference in KOOS‐12 between patients with no QTM and patients with QTM and greater than 2° of femoral component external rotation (80.3 [SD 16.1] vs. 79.3 [SD 18.1]). The comparison of KOOS subscale PASS percentages in patients with QTM and greater or less than 2° of femoral rotation is shown in Table 6.

**Table 5:** Comparison of PROMs in patients with QTM and less than or greater than 2° of femoral component external rotation.

	QTM with femoral rotation ≤2° (*n* = 37)		QTM with femoral rotation >2° (*n* = 33)		Mean difference	95% CI	*p* value
	Mean	SD	Mean	SD			
**KOOS**							
Pain	77.6	16.4	86.9	15.6	9.3	1.7–17.0	0.009
Symptoms	71.4	16.4	79.6	16.2	8.2	0.3–16.2	0.021
ADL	78.7	16.4	87.4	16.5	8.7	0.9–16.6	0.015
Sport	49.3	27.1	56.5	26.4	7.2	−5.6–20.0	NS
QoL	61.0	19.9	72.1	21.4	11.2	1.3–21.0	0.013
KOOS‐12	70.0	16.8	79.3	18.1	9.3	1.0–17.6	0.015
**Change in KOOS‐12**	**31.3**	**21.3**	**43.0**	**21.9**	**11.7**	**1.4–22.0**	**0.013**
**FJS**	48.0	28.9	58.8	30.6	10.8	−3.5–25.1	NS
**EQ‐VAS**	74.9	21.9	77.6	20.8	2.7	−7.6–13.0	NS

Abbreviations: ADL, activities of daily living; CI, confidence interval; EQ‐VAS, EuroQol Visual Analogue Scale; FJS, Forgotten Joint Scale; KOOS, Knee Injury and Osteoarthritis Outcome Score; PROM, patient‐reported outcome; QoL, quality of life; QTM, quadriceps tendon malalignment.

**Table 6:** Comparison of the KOOS PASS percentage in patients with Quadriceps Tendon Malalignment (QTAx > 14°) and greater or less than 2° of femoral component external rotation relative to the preoperative posterior condyles.

	Femoral rotation ≤2° (*n* = 40)	Femoral rotation >2° (*n* = 36)	*p* value
**KOOS PASS**	%	%	
Pain (>84.5)	37.8	69.7	0.007
Symptoms (>80.5)	25.0	56.3	0.008
ADL (>83.0)	43.2	78.8	0.002
QoL (>66.0)	40.5	63.6	0.045

Abbreviations: ADL, activities of daily living; KOOS, Knee Injury and Osteoarthritis Outcome Score; PASS, patient acceptable symptom state; QoL, quality of life; QTAx, quadriceps tendon axial angle.

## DISCUSSION

The key findings of this study are, first, that the QTM is a risk factor for significantly poorer PROMs following TKA and second, in patients with QTM, additional femoral component external rotation is associated with improved outcomes.

When considering the overall impact of QTM on PROMs following TKA, lower reported scores in patients with QTM were statistically significant but not clinically significant. For example, the average difference in KOOS‐12 at 12 months postoperatively was 7 points lower in patients with QTM compared to non‐QTM patients. However, the MCID for KOOS‐12 is considered to be 11.1 [7] making this difference clinically insignificant. This was common across other PROMs including the KOOS subscales, the FJS and EQ‐VAS.

The second phase of this study examined the effect of femoral component rotation on PROMs in patients with QTM. This showed an association between increased femoral component external rotation and PROMs in patients with QTM. Correlation analysis demonstrated a moderately strong association (*r *= 0.345 to 0.397) between femoral component rotation and KOOS‐12, FJS and pre‐ and postoperative change in KOOS‐12. A strong correlation would require a Pearson's correlation of >0.5. The scatter graph (Figure 2) shows that while there were still some patients with QTM and less femoral component rotation who did well, the likelihood of a good outcome increased significantly in patients with more than approximately 2° of external rotation.

Patients with QTM were analysed in three ways to attempt to quantify the effect of femoral component rotation on PROMs. First, applying a KOOS‐12 cut‐off score based on the median score of 75 points, patients with QTM were divided into two approximately equal groups. The group with better PROMs were noted to have a higher mean femoral component external rotation. Further analysis of this subgroup showed that there was a significant relationship (*r *= 0.536, *p *< 0.001) between the preoperative QTAx angle and the amount of femoral component external rotation required to achieve inclusion in the group of good outcome patients with a score of >75 points (Figure 3). This suggests a dose‐dependent relationship between the preoperative QTM and the amount of femoral component rotation required to achieve superior PROMs. Second, the same group of patients with QTM was divided into two approximately equal groups based on femoral rotation of greater or less than the median score of 2° from the preoperative posterior condyles. There was a statistically and clinically significant difference in KOOS‐12 scores (11.7 points) benefitting the group with more than 2° of externally rotation. This suggests QTM pathophysiology can be corrected or accommodated by altering the flexion‐extension axis of the knee to compensate for the extensor mechanism deformity. Finally, the PASS percentages were calculated on the patients with more or less than 2° of femoral component external rotation. This confirmed a significant improvement in the PASS percentages for the subscales of the KOOS score in patients with QTM who had >2° of femoral external rotation (Table 6).

Current concepts in knee arthroplasty alignment technique generally assume the extensor mechanism is always orientated perpendicular to the flexion‐extension axis of the knee or the axial alignment of the trochlear groove, or both [8, 14, 15, 25, 26]. Based on these assumptions, the aim of personalised alignment techniques is to recreate the native flexion–extension axis of the knee and the alignment of the trochlear groove. These assumptions cannot be maintained if the vector force of the quadriceps muscle is highly variable and not linked to the alignment of the trochlear groove [27]. There is evidence that lateralisation relative to the mechanical axis and the trochlear groove (QTCA), or external rotation of the quadriceps tendon relative to the shaft of the femur (QTAx), leads to a mismatch between the alignment of the extensor mechanism and both the flexion–extension axis of the knee and the alignment of the trochlear groove in a subgroup of patients [27]. QTM was shown to be a pathological deformity of the extensor mechanism by the paper from Talbot et al. which showed that it was closely linked to the development of lateral facet patellofemoral osteoarthritis. This deformity will be recreated if the native anatomy is recreated during TKA leading to a recreation of the native patella maltracking. Mizuno et al. recognised the importance of the force vectors related to osseous anatomy leading to patellar maltracking and altered tibiofemoral kinematics [21]. Biomechanical studies have shown the influence of femoral component rotation on patellar maltracking [16], quadriceps weakness and impaired gait [23].

The pull of the quadriceps tendon can, therefore, be seen as an independent soft tissue factor altering biomechanics and influencing outcomes after TKA. The current results reinforce this, demonstrating the outcomes of TKA are both statistically and clinically, significantly worse in patients with QTM if the position of the posterior condyles is recreated. These results contradict the idea that recreating the native flexion–extension axis or trochlear groove anatomy is the best approach in every patient and suggest that patients with preoperative anatomical deformity, such as QTM, changing the native anatomy to correct or accommodate deformity may be an advantage. This individualised approach provides a potential advancement to the previous paradigm of optimal femoral component alignment being parallel posterior condylar axis [6] or to the surgical epicondylar axis [28]. Future advancements in individualised alignment may involve the analysis of multiple anatomical factors such as the extensor mechanism alignment to determine the best component position for any individual combination of anatomical variations. This will require large databases with preoperative, intraoperative and postoperative measurements combined with accurate patient outcomes. It is likely to also require accurate assistive technologies to implement the individualised plans intraoperatively.

While 19.6% of patients in this study had a degree of quadriceps malalignment which appeared to affect their outcomes, the remaining 80.4% of patients did not. There was no difference in the outcomes of patients with neutral QTAx (<15°) and those with severely medialised quadriceps tendons. This would suggest that approximately 80% of patients do not benefit from additional femoral component external rotation. These patients are likely to be best served by alignment philosophies such as kinematic alignment, functional alignment and inverse kinematic alignment which prioritise ligament balance and which lead to femoral component rotation closer to the posterior condylar axis. Previous studies which have not found a difference in outcomes between kinematic alignment and mechanical alignment may have been influenced by the inclusion of patients with preoperative extensor mechanism malalignment [31]. This subgroup of patients may have benefitted from the additional femoral component external rotation provided by mechanical alignment.

The next evolution of personalised alignment techniques for TKA should identify patients with pathological extensor mechanism alignment and alter the component position commensurate with the severity of malalignment. An algorithm to guide the rotation of the femoral component could be developed which may vary between component types due to variations in design such as trochlear groove alignment and lateralisation, anterior femoral offset, trochlear ridge height, and tibiofemoral stability. Additional surgical factors also need to be considered for their effect on patella maltracking. These include changes in alignment such as tibial component rotation, joint‐line obliquity, changes in the anterior offset of the femoral component, patella component position, the amount of bone removed by the lateral facetectomy and the angle and depth of patella resection, and changes in surgical technique including lateral retinacular release and surgical approach. Alterations in femoral component rotation will also require adjustments to coronal and sagittal component position to obtain adequate ligament balance within a safe envelope.

This research has limitations, including a lack of randomisation. Femoral component position was determined intraoperatively and based on a combination of factors including the preoperative posterior condylar angle on CT scan, trochlear groove alignment, coronal alignment, ligament balance and the presence of severe patella maltracking. While coronal component alignment, tibial rotation and change in coronal component alignment were shown to have no effect on PROMs in patients with QTM, other factors such as patella resection, anterior femoral offset and component lateralisation were not measured. This has led to a wide range of component rotations which allowed for comparative analysis but may introduce undetermined confounding factors. Due to the retrospective nature of this study, QTA was not measured at the time of surgery and, therefore, did not influence the choice of alignment. The study was sufficiently powered to conduct the predetermined analyses. However, a larger sample is required to establish the optimal positioning of the femoral component for any given degree of extensor mechanism malalignment.

## CONCLUSION

QTM is a predictor of patella maltracking and an risk factor for poor patient outcomes following TKA. Adjustment to the flexion–extension axis of the knee by externally rotating the femoral component relative to the native posterior condyles led to improved outcomes in this subgroup of patients.

## AUTHOR CONTRIBUTIONS

Simon Talbot designed the study, collected data and wrote the manuscript. Rachel Zordan analysed data and wrote the manuscript. Francesca Sasanelli collected data and wrote the manuscript. Matthew Sun collected data and wrote the manuscript. All authors read and approved the final manuscript.

## CONFLICT OF INTEREST STATEMENT

The authors declare no conflicts of interest.

## ETHICS STATEMENT

Ethics approval was sought and gained from the St. Vincent's Health Network (HREC/18/SVH/250). Informed consent was obtained from all participants to prospectively collect and analyse scans and outcome data.

## Data Availability

The data that support the findings of this study are available on request from the corresponding author. The data are not publicly available due to privacy or ethical restrictions.
